# Betaxanthin Profiling in Relation to the Biological Activities of Red and Yellow *Beta vulgaris* L. Extracts

**DOI:** 10.3390/metabo13030408

**Published:** 2023-03-09

**Authors:** Aneta Spórna-Kucab, Anna Tekieli, Agnieszka Grzegorczyk, Łukasz Świątek, Anastazja Boguszewska, Krystyna Skalicka-Woźniak

**Affiliations:** 1 Department of Chemical Technology and Environmental Analytics, Faculty of Chemical Engineering and Technology, Cracow University of Technology, Warszawska 24, 31-155 Krakow, Poland; 2 Department of Pharmaceutical Microbiology, Faculty of Pharmacy, Medical University of Lublin, Chodźki 1, 20-093 Lublin, Poland; agnieszka.grzegorczyk@umlub.pl; 3 Department of Virology with SARS Laboratory, Faculty of Medicine, Medical University of Lublin, Chodźki 1, 20-093 Lublin, Poland; lukasz.swiatek@umlub.pl (Ł.Ś.); anastazja.boguszewska@umlub.pl (A.B.); 4 Department of Natural Products Chemistry, Medical University of Lublin, Chodźki 1, 20-093 Lublin, Poland; kskalicka@pharmacognosy.org

**Keywords:** antimicrobial, cytotoxicity, anticancer, metabolites, betaxanthins, natural pigments, *Beta vulgaris* L., LC-MS

## Abstract

*Beta vulgaris* L. is an edible plant with health-beneficial activities. The profile of betaxanthins is more complex than previously described in beetroot cultivars. Twenty-four betaxanthins were detected in extracts of the peel and flesh of five cultivars by HPLC-DAD-ESI-MS, of which two new betaxanthins (arginine-Bx and ornithine-Bx) were detected for the first time in *B. vulgaris* cultivars. The content of betaxanthins in the studied cultivars decreased in the Tytus > Ceryl > Chrobry > Forono > Boldor sequence. The highest content of compounds (1231 mg/100 g DE) was observed in the Tytus cultivar (peel). The peel of *B. vulgaris*, which is often considered a waste, appeared to be a richer source of betaxanthins compared to its flesh. Antibacterial and antifungal activities were determined against twenty-three microorganisms. Tytus (peel) showed a moderate or good bactericidal effect, especially against the majority of Gram-positive bacteria as well as against most of the tested fungi (MIC = 0.125–0.5 mg/mL) and additionally characterized by low cytotoxicity towards non-cancerous cells (CC_50_ = 405 μg/mL, CC_50_—50% cytotoxic concentration). Tytus flesh also showed a high cytotoxicity value against human cervical adenocarcinoma (HeLa), with CC_50_ of 282 μg/mL. Correlation analysis was used to determine the relationship between the betaxanthin profiles and antimicrobial and anticancer activities. Arginine-Bx, proline-Bx, and tryptophan-Bx were indicated as active against HeLa and the colon cancer cell line (RKO), while asparagine-Bx and phenylalanine-Bx was responsible for activity against all tested bacterial and yeast species. The significant effectiveness and safety of these beetroots make indicated compounds promising applicants as antimicrobial and anticancer agents.

## 1. Introduction

Numerous natural compounds isolated from plant material are screened in order to check their pharmacological activities. Extensive research on these compounds demonstrates that they exhibit a wide spectrum of actions against human diseases as well as biological activities [1,2,3,4,5].

One of the plants with health-beneficial properties is beetroot (*Beta vulgaris* L.), usually cultivated as a vegetable with edible roots and leaves [6,7,8]. Recently, we have noticed an increase in interest in beetroot, especially due to its biological activity, including its positive effects on gastrointestinal health [7,9]. The consumption of beets supports the treatment of many diseases due to the presence of compounds that are very strong antioxidants and have anti-inflammatory effects [7]. The health-promoting effect of beets is often associated with the presence of betalains (betacyanins and betaxanthins), which have shown anti-inflammatory, antioxidant, chemopreventive [7,10], antimicrobial [11], antimalarial actions [12,13,14,15,16], as well as hepatoprotective, hypolipidemic and neuroprotective activities [14,15,16].

**Figure 1:**
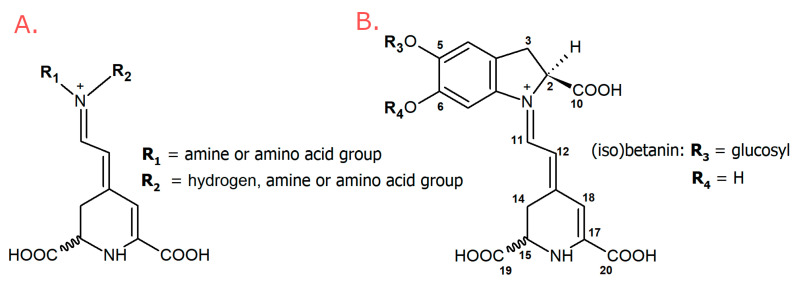
General structures: (**A**) betaxanthins, (**B**) betacyanins.

Betalains are derived from tyrosine via the immonium conjugates of betalamic acid with *cyclo*-DOPA (red-violet betacyanins) and amino acids or amines (yellow-orange betaxanthins) (Figure 1) [17].

The simplest betacyanin is betanidin (betalamic acid + *cyclo*-DOPA). The most widespread betacyanin among plants is betanidin-5-*O*-*β*-glucoside, commonly named betanin [17,18,19,20,21,22]. Betaxanthins are condensation products of betalamic acid with amines or amino acids. The most common betaxanthins are vulgaxanthin I (glutamine-Bx) and indicaxanthin (proline-Bx), found in yellow beet and cactus pear, respectively. Different amino acid or amine side chains determine the color of betaxanthins [12,23].

More than 90 betalains (60 betacyanins and 33 betaxanthins) have been reported to occur naturally in plants of approximately 17 families within the order Caryophyllales [15,24,25]. Most plants contain mainly betacyanins, but in some sources, such as yellow beet, cactus pear, and *Portulaca grandiflora*, betaxanthins are the dominant pigments [9,10,11,26,27]. The yellow or red beet color is influenced by both the total amount and the prevalence of different betalains (betaxanthins or betacyanins). White beet has overall very low levels of betalains [28,29].

The dominance of betacyanins in the world of plants may be associated with much greater interest in these compounds [11,12,18,19,23,30]. Betaxanthins can be synthesized, which significantly increases their possibility of application in the future; however, the synthesis of selected betaxanthins requires the development of appropriate procedures [31,32]. Therefore, plants containing a variety of betaxanthins are a valuable source for gaining those bioactive compounds.

This report aims to explore the correlations between the betaxanthin profiles in yellow and red *B. vulgaris* (flesh and peel) and the antimicrobial and anticancer properties they exhibit. To the best of our knowledge, so far, the comprehensive betaxanthin profiles in different varieties of *B. vulgaris* as well as their potential antimicrobial and anticancer activities, have not been reported.

## 2. Materials and Methods

### 2.1. Plant Material

The experiments were conducted on the red beetroot cultivars: Ceryl, Chrobry, Forono, and Tytus, as well as the yellow cultivar Boldor. Red *B. vulgaris* were harvested by the Spójnia company (Nochowo, Poland), while the yellow cultivar was by the Bejo company (Ożarów Mazowiecki, Poland) in 2019. The fresh beetroots were washed under running water and peeled to obtain the peel and flesh. Then the peel and flesh were weighted separately before extraction according to a procedure described in Section 2.3.

### 2.2. Reagents and Reference Compounds

For the identification of betaxanthins, authentic standards from previous betalain studies were used [33] or semi-synthesized [34].

Ethyl alcohol pure p.a. was purchased from Avantor Performance Materials Poland S.A. (Gliwice, Poland). LC-MS-grade methanol and formic acid (purity ≥ 98%) were obtained from Sigma-Aldrich (St. Louis, MO, USA).

Reference strains of bacteria and fungi from the American Type Culture Collection (ATCC, LGC Standards, Teddington, UK) and Centers for Disease Control and Prevention (CDC, Atlanta, GA, USA) were used in the study. These strains included nine Gram-positive bacteria (*Staphylococcus aureus* ATCC 25923, and 29213—methicillin-sensitive strains, *Staphylococcus aureus* ATCC 43300 and BAA-1707—methicillin-resistant strains, *Staphylococcus epidermidis* ATCC 12228, *Enterococcus faecalis* ATCC 29212, *Micrococcus luteus* ATCC 10240, *Bacillus subtilis* ATCC 6633, *Bacillus cereus* ATCC 10876,), six Gram-negative bacteria (*Salmonella* Typhimurium ATCC 14028, *Proteus mirabilis* ATCC 12453, *Bordetella bronchiseptica* ATCC 4617, *Escherichia coli* ATCC 25922, *Klebsiella pneumoniae* ATCC 13883, *Pseudomonas aeruginosa* ATCC 27853), and eight fungal species belonging to the genus *Candida* (*C. parapsilosis* ATCC 22019, *C. albicans* ATCC 10231 and 2091, *C. glabrata* ATCC 90030, *C. krusei* ATCC 14243, *C. auris* CDC B11903, *C. lusitaniae* ATCC 3449, *C. tropicalis* ATCC 1369).

Cell lines used for in vitro experiments included VERO (ATCC, CCL-81, monkey kidney), HeLa (ATCC, CCL-2, human cervical adenocarcinoma), and RKO (ATCC, CRL-2577, human colon cancer). The cells were cultured using MEM (cancer cells) or DMEM (VERO) media (Corning, Tewksbury, MA, USA) containing penicillin–streptomycin solution (Corning). The phosphate-buffered saline (FBS) and trypsin were obtained from Corning, while fetal bovine serum (FBS) was from Capricorn Scientific (Ebsdorfergrund, Germany). Sodium dodecyl-sulfate (SDS) was acquired from PanReac Applichem (Darmstadt, Germany), dimethylformamide (DMF), and dimethyl sulfoxide (DMSO, p.a.) from Avantor Performance Materials (Gliwice, Poland), while 3-(4,5-dimethylthiazol-2-yl)-2,5-diphenyltetrazolium bromide (MTT) from Sigma-Aldrich (St. Louis, MO, USA).

### 2.3. Sample Preparation

The 100 g sample (fresh peels and flesh from five *Beta vulgaris* L. cv.) was ground into small pieces using a blender (thermomix, Vorwerk, Wuppertal, Germany) and subjected to maceration three times for 30 min using 300 mL of 80% ethanol at room temperature each time. Three extractions were performed for each cultivar. Obtained extracts were transferred to the volumetric flask, and the solutions were made up to 1 L. Next, 200 μL of every extract was centrifuged at 4000 rpm for 5 min, and quantitative analysis of total betaxanthin was performed according to a procedure described in Section 2.4.

The remaining extracts were partially evaporated at 25 °C under reduced pressure (rotary evaporator, Heidolph, Schwabach, Germany) and frieze-dried (Christ, Osterode am Harz, Germany). The dried extracts were weighed and used in further research on their betaxanthin profiles (Section 2.5).

### 2.4. Quantitation and Qualitation of Compounds

The content of betaxanthins in the fresh peels and flesh of all cultivar extracts dissolved in the extraction solvents was determined spectrophotometrically with a microplate reader (Tecan Infinite 200, Grödig, Salzburg, Austria), respectively, according to the methods of Stintzing et al. [35]. Measurements of the absorption values for the extracts (200 μL) were performed in the range of λ 300–700 nm with a wavelength step of 1 nm step at 25 °C in triplicate. The absorbance reading was used to calculate the betaxanthin content of each sample. The betaxanthin content (BC) was calculated as BC (mg/L) = ([A × DF × MW × 1000]/[e × l]), where A is the absorption, DF is the dilution factor, and l is the path length (0.53 cm) of the microplate. For the quantification of betaxanthins, the molecular weights (MW = 339 g/mol) and molar extinction coefficients (e = 48.000 cm^−1^ mol^−1^ L) were applied. Fresh extracts (each 1 L) were dried and weight. The betaxanthin content was expressed as mg of pigment in 100 g of dried flesh or peel extracts of *B. vulgaris*.

The quantitative determination of single betaxanthins was estimated from the peak areas using MS chromatograms of studied *B. vulgaris* extracts. *P. grandiflora* extract [33], including 19 identified betaxanthins, was used as the reference standard for the description of individual betaxanthins in *B. vulgaris* extracts. All samples (30 mg/1 mL) were analyzed in triplicate. For the evaluation of the instrumental precision for each sample, three independent LC-MS runs were conducted. It was shown that with repetition of the same sample (n = 3), the relative standard deviation was 6.3–7.4%.

### 2.5. HPLC-MS Analysis

The qualitative and quantitative determination of single betaxanthins in yellow and red *B. vulgaris* were determined by HPLC-DAD-ESI-MS (LCMS-8030 system, Shimadzu, Japan). Quantitative analysis was performed as previously described [33]. The LC-MS system consisted of SIL-20ACXR autosampler, degasser, and LC-20ADXR Nexera binary pump. Samples were eluted through a 100 mm × 4.6 mm i.d., 5.0 μm Kinetex C_18_ chromatographic column (Phenomenex, Torrance, CA, USA) protected by a 4 mm × 2 mm, i.d., 5.0 μm guard column of the same material (Phenomenex). The column was thermostated at 40 °C.

All samples before analysis were diluted in demineralized water and centrifuged at 4000 rpm for 5 min. The analyses were performed with a binary gradient. The mobile phase was composed of methanol (A) and 2% aqueous formic acid (B). A flow rate of 0.5 mL/min was used, and 15 μL of the sample was injected. The solvent gradient system for extracts was: 1% A in B at 0 min, a gradient to 11% A in B at 12.0 min and 60% A in B at 24 min, then a gradient to 90% A in B at 24.01 min. The UV/Vis spectra were collected using a DAD detector.

In ESI-MS experiments, the capillary voltage used in the positive electrospray ionization mode was 4.5 kV at a capillary temperature of 250 °C. ESI-MS data were recorded using scan mode with *m/z* ranging from 100 to 2000 Da and selected ion monitoring (SIM). For data acquisition in HPLC-DAD-ESI-MS, the LabSolutions version 5.91 SP1 software was used.

### 2.6. Antimicrobial Activity

The tests were performed using the microdilution broth method according to the guidelines of the European Committee on Antimicrobial Susceptibility Testing (EUCAST) [36]. The minimum inhibitory concentration (MIC) of the selected extracts was determined for twenty-three reference strains of the American Type Culture Collection (ATCC). All the microbial strains used were first subcultured on Mueller-Hinton Agar for bacteria or RPMI 1640 Agar for yeasts and incubated at 35 °C for 24 h (bacteria) and 30 °C for 24 h (fungi). Microbial colonies were collected and suspended in sterile physiological saline to obtain an inoculum of 0.5 McFarland standard, corresponding to 1.5 × 10^8^ CFU/mL (colony forming units) for bacteria and 5 × 10^6^ CFU/mL for yeasts. The extracts were dissolved in pure DMSO to obtain the final concentration of 500 mg/mL. The concentration of DMSO was 6.4% in the final stock solution in Mueller-Hinton Broth (MHB) or RPMI 1640 medium at a concentration of extracts of 32 mg/mL and decreased two-fold with each serial dilution. The two-fold dilutions of extracts in MHB for bacteria or RPMI 1640 for fungi were prepared in 96-well polystyrene plates to obtain final concentrations tested were 32, 16, 8, 4, 2, 1, 0.5, 0.25, 0.125, 0.06 and 0.03 mg/mL. At the same time, tests were always performed: control of the sterility of the medium (MHB and RPMI 1640), control of the viability of bacterial and fungal strains, control of extracts, and control of DMSO. The antimicrobial studies were performed as previously described [37]. To determine the MIC (minimum inhibitory concentration), the absorbance was measured in a spectrophotometer at a wavelength of 600 nm. Minimum bactericidal concentration (MBC) or minimum fungicidal concentration (MFC) was also determined. The experiments were performed in triplicate. Based on each MIC, MBC, and MFC value, the most common representative value, i.e., mode, was presented. The standard chemotherapeutics agents: vancomycin (range of 0.06–16 µg/mL), ciprofloxacin (range of 0.015–16 µg/mL), and fluconazole (range of 0.06–16 µg/mL) were used as antimicrobial substances active against Gram-positive bacteria, Gram-negative bacteria, and fungi, respectively.

### 2.7. Cytotoxicity Evaluation and Anticancer Selectivity

Cell line subculturing and experiments were performed at 37 °C in the 5% CO_2_ atmosphere (CO_2_ incubator, Panasonic Healthcare Co., Ltd., Tokyo, Japan). The extracts of *B. vulgaris* were dissolved (100 mg/mL) in DMSO to obtain the stock solution for the evaluation of cytotoxicity.

The cytotoxicity was tested using the microculture tetrazolium assay as previously described [38]. Briefly, cell monolayers in 96-well plates were treated for 24 h with serial dilutions of test extracts. After the incubation, the media was removed, plates were washed with PBS, MTT-supplemented media was added, and incubation continued for another 4 h. The precipitated formazan crystals were then dissolved using SDS/DMF/DMSO, and after overnight incubation, the absorbance (540 and 620 nm) was tested using Synergy H1 Multi-Mode Microplate Reader (BioTek Instruments, Inc., Winooski, VT, USA). Data were analyzed using GraphPad Prism (version 7.04), and concentrations reducing the cellular viability by 50% (CC_50_) were calculated from dose-response curves (curve fit–nonlinear regression). Moreover, the anticancer selectivity was evaluated by calculating the selectivity indices (SI) (SI = CC_50_VERO/CC_50_CancerCells, SI > 1 suggests anticancer selectivity).

### 2.8. Statistical Evaluation

The data are presented as mean ± standard deviation (SD) for triplicate analysis. Results were statistically analyzed with Statistica version 7.1 (StatSoft, TIBCO Software Inc., Palo Alto, CA, USA), using one-way variance analysis (ANOVA) of the average of five cultivars of *B. vularis* (Ceryl, Chrobry, Forono, Tytus, and Boldor). The results were analyzed by ANOVA and Tukey’s Post Hoc test to determine the differences between samples. Significance was assessed at α level 0.05 to find out how many and which *B. vulgaris* cultivars have different contents. *p*-values less than 0.05 were considered statistically significant. The statistical analysis of correlations was done with Pearson’s test for significance of correlation coefficient.

## 3. Results and Discussion

### 3.1. The Profile and Content of Betaxanthins in B. vulgaris

Currently, the most widespread and rich source of betalains is red beet, which the composition of betacyanins has been thoroughly characterized [23,39,40,41,42,43,44]. On the contrary, the profile of betaxanthins in red and yellow beetroots has been described only to a small extent in the literature [34,39,40,42,45].

**Figure 2:**
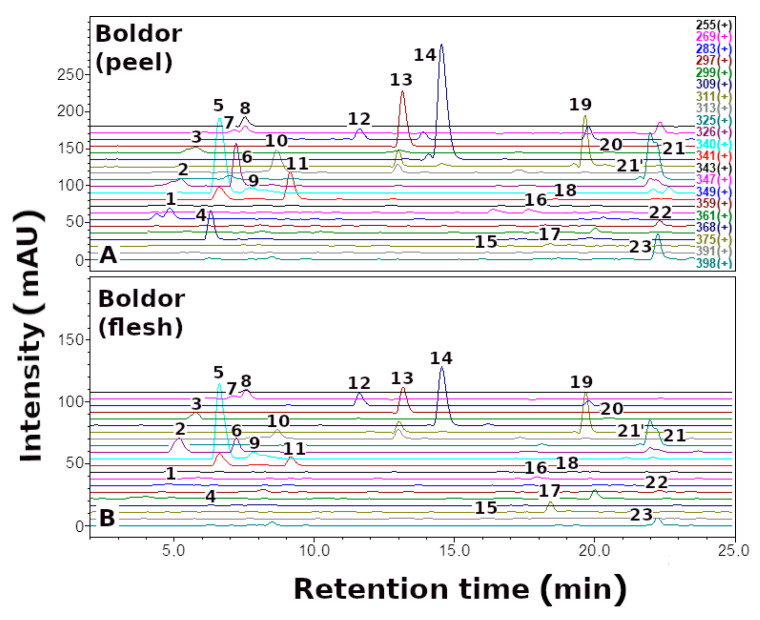
Selected-ion monitoring chromatogram (ESI-MS) in positive ion mode for betaxanthins of yellow *Beta vulgaris* L.: (**A**) peel, and (**B**) flesh. Numbers and names are available in Table 1.

**Figure 3:**
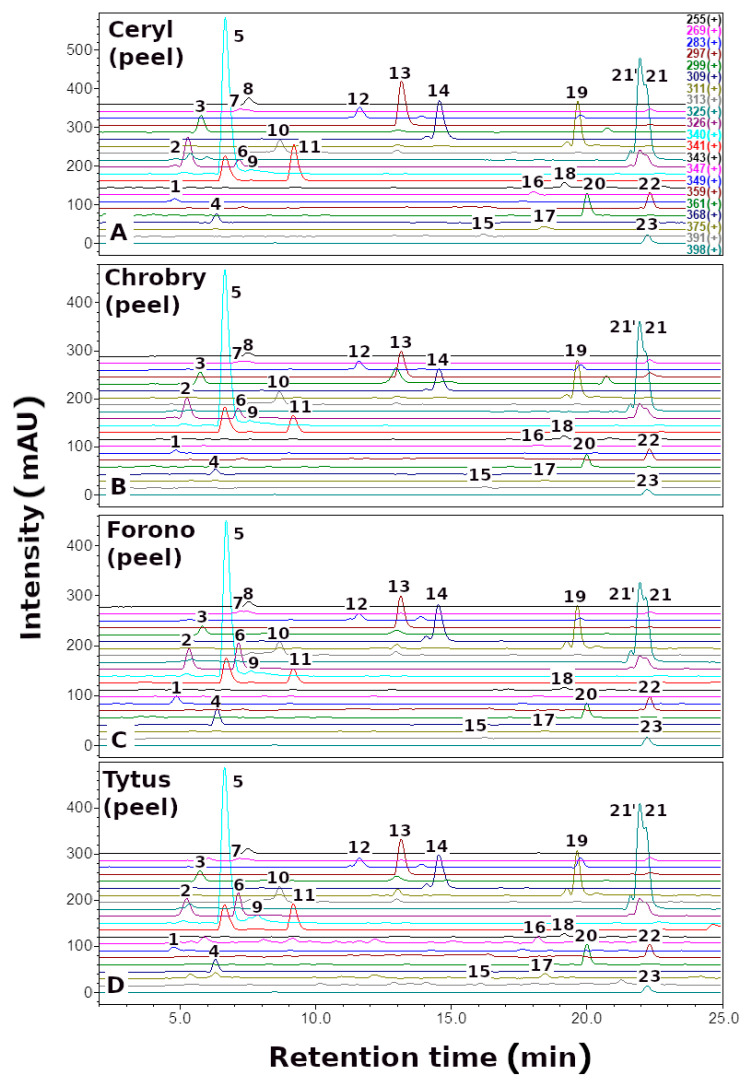
Selected-ion monitoring chromatogram (ESI-MS) in positive ion mode for betaxanthins of red *Beta vulgaris* L. peels: (**A**) Ceryl, (**B**) Chrobry, (**C**) Forono, and (**D**) Tytus. Numbers and names are available in Table 1.

**Figure 4:**
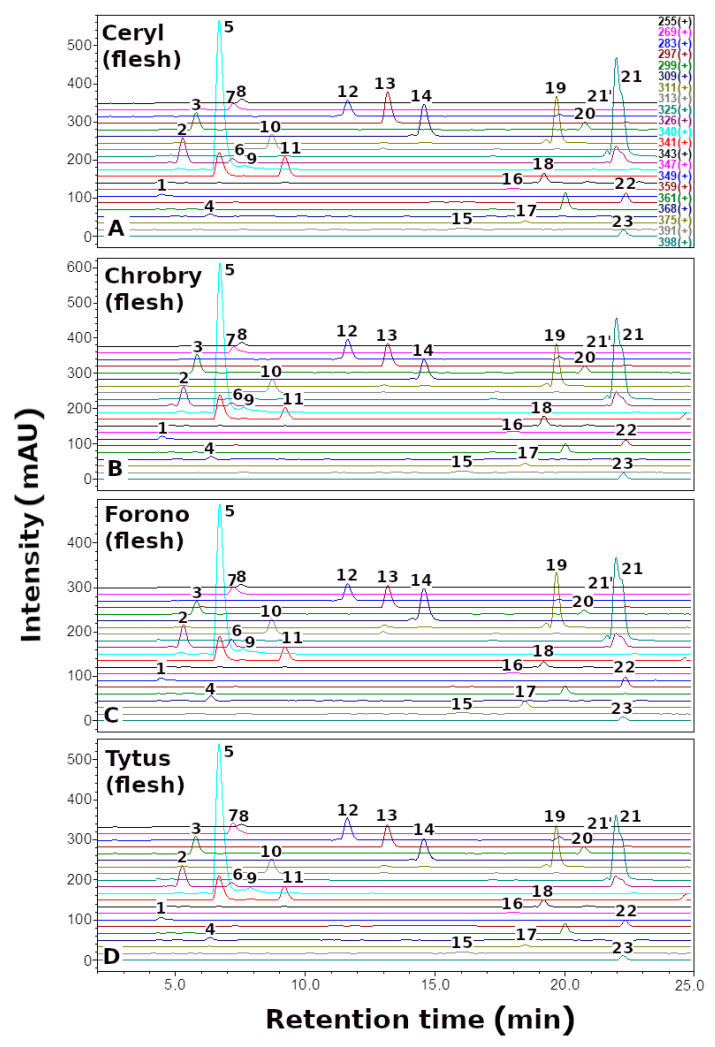
Selected-ion monitoring chromatogram (ESI-MS) in positive ion mode for betaxanthins of red *Beta vulgaris* L. flesh: (**A**) Ceryl, (**B**) Chrobry, (**C**) Forono, and (**D**) Tytus. Numbers and names are available in Table 1.

Here, we report the quantitative and qualitative (Figure 2, Figure 3 and Figure 4, Table 1) profile of betaxanthins of the peel and flesh extracts of four red (Ceryl, Chrobry, Forono, Tytus) and one yellow (Boldor) *Beta vulgaris* L. cultivars. These are popular edible beet cultivars.

The highest concentration of betaxanthins was noticed in the red variety Tytus peel (1231 mg/100 g Dry Extract (DE)), while the yellow variety Boldor flesh accumulated the lowest content of betaxanthins (317 mg/100 g DE). It might seem that yellow beetroot reflecting the yellow-orange color of betaxanthins will be their richer source than red beetroot. However, it is worth noting that in red beets, the yellow-orange betaxanthins are visually overpowered by the red-violet betacyanins, reflecting the color of the red beet but not its actual composition. The total concentration of betaxanthins in the extracts of *B. vulgaris* decreased in the following order, depending on the cultivar: Tytus > Ceryl > Chrobry > Forono > Boldor.

The peel of *B. vulgaris*, which is often considered a waste, appeared to be a richer source of betaxanthins compared to its flesh [46]. The highest concentration of betaxanthins in the peel extracts of *B. vulgaris* was observed in Tytus (1231 mg/100 g DE), while the lowest was noticed in Boldor (574 mg/100 g DE). In contrast, the lowest concentration of these pigments in *B. vulgaris* flesh extracts was also recorded in Boldor (317 mg/100 g DE) and the highest was in Chrobry (609 mg/100 g DE) (Table 2 and Table 3).

In addition to *B. vulgaris*, *Portulaca grandiflora* Hook. is also a proven rich source of betaxanthins. Our research team [33] investigated the quantitative and qualitative profile of betaxanthins from yellow, orange, red, and purple flowers of *P. grandiflora*. The content of betaxanthins decreased according to the following flower color order: orange > yellow > purple > red (982, 417, 323, and 162 mg/100 g DE, respectively) [33]. It should be noted that a comparable source of these pigments to the peel extract of the Ceryl cultivar (919 mg/100 g DE) is the orange flower extract of *P. grandiflora* (982 mg/100 g DE).

Qualitative analysis of *B. vulgaris* extracts by LC-DAD-ESI-MS showed the presence of 24 yellow-orange betaxanthins. The compounds identified, as well as their retention times, λ_max_ values, and (M + H)^+^ data, are summarized in Table 1. Reference compounds semi-synthesized [34] or isolated of *P. grandiflora* fruit extracts [23] were used to identify betaxanthins.

Betaxanthins analysis in *B. vulgaris* peel and flesh extracts revealed an identical profile in red (Ceryl, Chrobry, Forono, and Tytus) and yellow (Boldor) cultivars; however, the profile of betaxanthins is more complex than previously described in yellow beetroot (Boldor) [42] and other varieties of *B. vulgaris* [39,40,45], comprising additionally arginine-Bx (**4**) and ornithine-Bx (**6**).

Kugler et al. [40] also identified a total of 24 betaxanthins in the yellow Boldor beet variety, including tyramine-Bx and aspartic-Bx, which were not identified in our study [40]. Our research team [33] identified 19 betaxanthins in *P. grandiflora* that are included in the *B. vulgaris* cultivars tested, with the exception of histamine-Bx [33]. It can be concluded that the tested cultivars of *B. vulgaris* are a much richer source of betaxanthins than the inedible *P. grandiflora*.

**Figure 5:**
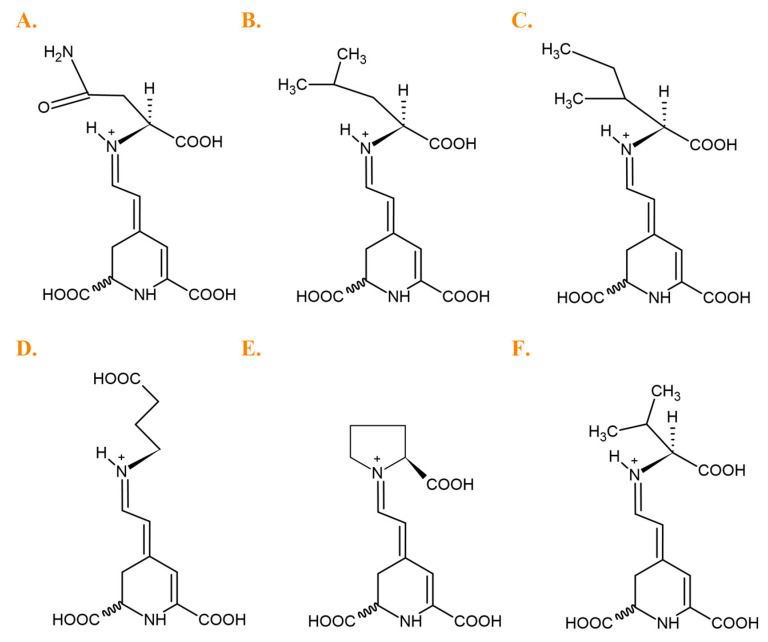
Predominant betaxanthins detected in *Beta vulgaris*, L. cultivars: (**A**) glutamine-Bx (**5**), (**B**) leucine-Bx (**21**), (**C**) isoleucine-Bx (**21′**), (**D**) *γ*-aminobutyric acid-Bx (**13**), (**E**) proline-Bx (**14**), (**F**) valine-Bx (**19**).

The dominant compounds in the *B. vulgaris* cultivars were glutamine-Bx (**5**), isoleucine-Bx (**21′**), leucine-Bx (**21**), *γ*-aminobutyric acid-Bx (**13**), proline-Bx (**14**) and valine-Bx (**19**) (Figure 5).

Definitely, the extract obtained from the peel of the Tytus variety is the best source of dominant compounds such as glutamine-Bx (**5**) (350.82 mg/100 g DE), isoleucine-Bx (**21′**) (159.96 mg/100 g DE), leucine-Bx (**21**) (150.47 mg/100 g DE), *γ*-aminobutyric acid-Bx (**13**) (70.06 mg/100 g DE) and valine-Bx (**19**) (74.29 mg/100 g DE), while yellow beet Boldor proline-Bx (**14**) (140.95 mg/100 g DE). The second best source of the above compounds is the extract obtained from the peel of the red beet of the Ceryl cultivars.

### 3.2. Antimicrobial Activity

Results of the antibacterial analysis of extracts obtained from five varieties of beetroot—Ceryl, Chrobry, Forono, Tytus, and Boldor are presented in Table 4, Table 5 and Table 6. It can be seen that the extracts showed diverse activity against the tested reference bacteria (MIC = 0.06–32 mg/mL) and *Candida* species (MIC = 0.125–8 mg/mL). The most sensitive strain was *Micrococcus luteus* ATCC 10240 (MIC = 0.06–8 mg/mL). Overall, only Gram-positive bacteria were susceptible to *B. vulgaris* extracts. This is also confirmed by studies by other authors from Canada who write that Gram-positive bacteria generally show higher sensitivity to red beetroot than Gram-negative ones [47].

According to our observations, it should be noted that the Tytus (peel) extract showed the highest activity (MIC = 0.125–0.5 mg/mL) against all Gram-positive bacteria except for *Bacillus subtilis* ATCC 6633 and *B. cereus* ATCC 10876, but according to other authors, MIC values of 0.100–0.625 mg/mL indicated moderate activity [48]. The pulp extract of this variety showed moderate activity against *M. luteus* ATCC 10240 (MIC = 0.5 mg/mL).

The extract of the Ceryl variety (peel) also showed significant activity (MIC = 0.06–0.125 mg/mL) against *Staphylococcus aureus* ATCC 25923 and *M. luteus* ATCC 10240 and *B. subtilis* ATCC 6633. It is worth noting that also the extract of Chrobry (peel) showed good activity (MIC = 0.125–0.5 mg/mL) against all Gram-positive bacteria except *B. cereus* ATCC 10876 (Table 4). The similarity results had the other author, who tested the in vitro antibacterial activity of the ethanol extract of beetroot against the food pathogens: *S. aureus*, *B. cereus*, and *Escherichia coli* by two methods: disc diffusion method and microdilution methods using selected Gram-positive and Gram-negative [49]. According to their results, Gram-positive bacteria: *S. aureus* and *B. cereus* demonstrated higher susceptibility than Gram-negative—*E. coli*. Their extract showed antibacterial activity against *S. aureus* (MIC = 0.75 mg/mL), one of the most common Gram-positive bacteria causing food poisoning. On the other hand, weak antimicrobial activity was found against *E. coli* (MIC = 1.5 mg/mL) [49].

The peel extracts of the Tytus, Chrobry, and Ceryl varieties were the only ones that showed activity against most of the *Candida* species tested, with MIC values ranging from 0.125 to 1 mg/mL. *Candida parapsilosis* ATCC 22019, *C. albicans* ATCC 10231, and *C. albicans* ATCC 2091 were the most sensitive to Tytus and Ceryl extracts (MIC = 0.25 mg/mL), while *C. lusitaniae* ATCC 3449 and *C. tropicalis* ATCC 1369 were only susceptible to Tytus peel extract (MIC = 0.125–0.25 mg/mL) (Table 6). Tenor et al. [50] undertook to investigate the antifungal activity of beetroot against two *Candida* species, *C. albicans* ATCC 10231, *Rhizoctonia solani* ATCC 13048; four fungi: *Fusarium oxysporum* ATCC 695, *Cladosporium herbarum* ATCC 11281, *Botrytis cinerea* ATCC 11542, *Aspergillus flavus* ATCC 15517. They showed, as in this work, that betalains-rich extracts from red pitahaya (*Hylocereus polyrhizus*) were active against fungi (*C. albicans*, *R. solani*) at 0.125–0.25 mg/mL and fungi (*F. oxysporum*, *C. herbarum*, *B. cinerea*, *A. flavus*) at 0.5 mg/mL. The results of these researchers indicated that the peel, which is inedible and waste from these plants, can be subjected to simple purification methods to obtain extracts and/or various fractions and can be further used for the preparation of health products and supplements, e.g., for food preservation.

As shown in Table 4, the extract of the Forono variety (peel) showed versatile activity against Gram-positive bacteria (MIC = 0.006–16 mg/mL) with the highest activity against *B. subtilis* ATCC 6633 and *B. cereus* ATCC 10876 with MIC = 0.06 mg/mL, one of the most common Gram-positive bacteria causing food poisoning. In addition, it showed activity against fungi with MIC values ranging from 0.5 to 4 mg/mL, with the highest activity against *C. parapsilosis* ATCC 22019 (MIC = 0.5 mg/mL) (Table 6).

On the other hand, extracts from the Boldor variety, both from the peel and pulp, showed weak activity against all tested microorganisms (MIC = 2–16 mg/mL). In general, extracts obtained from beetroot peels were characterized by higher antimicrobial activity compared to extracts obtained from the pulp. All extracts showed low activity against all Gram-negative bacteria, with MIC values ranging from 2 to 32 mg/mL (Table 5).

Antimicrobial substances are usually considered bactericidal or fungicidal if the MBC/MIC or MFC/MIC ratio is ≤4. If the MBC/MIC or MFC/MIC ratio is >4, antimicrobial substances are usually considered to be bacteriostatic or fungistatic [37]. Based on the results presented, it can be concluded that most of the beetroot extracts showed bactericidal activity (MBC/MIC = 1–4) against most bacteria and fungi, and the extracts of the Tytus, Chrobry, Ceryl, and Forono varieties showed bacteriostatic activity (MBC/MIC = 8–64) and fungistatic (MFC/MIC = 8–32) to selected microorganisms with low MIC values.

The MIC values for the reference antimicrobials were 1 µg/mL fluconazole for *C. albicans* ATCC 10231, 1 µg/mL vancomycin for *S. aureus* ATCC 29213 and 0.015 µg/mL ciprofloxacin for *E. coli* ATCC 25922.

### 3.3. Correlation between Phytochemical Composition and Antimicrobial Activity

Correlations between identified betaxanthins and antimicrobial activity were checked for all extracts obtained from five varieties of *B. vulgaris*. The values of the correlation coefficient (R) were calculated based on the obtained MIC values and the absolute peak area of each assigned peak from the chromatograms of the individual betaxanthins. A negative R-value refers to decreasing in the MIC parameters with an increase in the component peak value (negative correlation), which proves the positive impact on antimicrobial properties.

All identified betaxanthins showed a negative correlation against one strain of Gram-positive bacteria *S. epidermidis* ATCC 12228 and two strains of Gram-negative bacteria *P. mirabilis* ATCC 1,453 and *K. pneumoniae* ATCC 13883. *S. epidermidis* are natural pathogens found on human skin, increasingly resistant to antibiotics and contributing to the occurrence of infections [51]. *P. mirabilis* is the cause of 90% of Proteus infections and occurs mainly in people with weakened immune systems [52]. *K. pneumoniae* is the fourth most common cause of Gram-negative hospital-associated infections, including urinary tract infections, pneumonia, and wound infections [53]. Therefore, it is worth noting that the betaxanthins identified in *B. vulgaris* extracts inhibit the activity of the above bacterial strains to some extent and may be a good candidate for further research in this respect.

Asparagine-Bx (**2**), ethanolamine-Bx (**8**), and phenylalanine-Bx (**22**) exhibited negative correlation against all strains of Gram-positive bacteria and fungi, while asparagine-Bx (**2**), glutamine-Bx (**5**), threonine-Bx (**10**), isoleucine-Bx (**21′**), leucine-Bx (**21**) and phenylalanine-Bx (**22**) against all strains of Gram-negative bacteria. On the other hand, in relation to all tested strains of fungi, a negative correlation was also shown by compounds such as arginine-Bx (**2**), ornithine-Bx (**6**), glutamic acid-Bx (**11**), *γ*-aminobutyric acid-Bx (**13**), proline-Bx (**14**), dopamine-Bx (**16**), 3-methoxytyramine-Bx (**20**), isoleucine-Bx (**21′**), leucine-Bx (**21**), and tryptophan-Bx (**23**).

It is also worth mentioning that glutamic acid-Bx (**11**), *γ*-aminobutyric acid-Bx (**13**), and 3-methoxytyramine-Bx (**20**) exhibited negative correlation against all strains of Gram-positive bacteria except B. cereus ATCC 10876. In contrast, tryptophan-Bx (**23**) and proline-Bx (**14**) showed positive effects against all strains of Gram-positive bacteria except *M. luteus* ATCC 10240. The negative correlation against all strains of Gram-positive bacteria was also exhibited arginine-Bx (**2**) and ornithine-Bx (**6**) except *E. faecalis* ATCC 29212.

The dominant betaxanthins (**5**, **13**, **14**, **21′**, and **21**) in *B. vulgaris* extracts showed a positive effect against all Gram-negative bacteria or fungi except valine-Bx (**19**). Nevertheless, other identified betaxanthins also had a positive effect on microbial activity against selected microorganisms. This shows that probably the concentration of individual betaxanthins did not significantly affect their potency against microorganisms.

A positive correlation against all strains of Gram-positive bacteria was exhibited by histidine-Bx (**1**), glycine-Bx (**7**), alanine-Bx (**12**), and methionine-Bx (**18**) except *S. epidermidis* ATCC 12228 and *M. luteus* ATCC 10240. Histidine-Bx (**1**), glycine-Bx (**7**), and additionally tyrosine-Bx (**17**) also showed a positive correlation with all strains of Gram-negative bacteria except *P. mirabilis* ATCC 12453 and *K. pneumoniae* ATCC 13883. The above five betaxanthins (**1**, **7**, **12**, **17,** and **18**) and serine-Bx (**3**) also did not affect activity against all fungal strains.

Previously we showed six betaxanthins from *Portulaca grandiflora* Hook., such as glutamine-Bx (**5**), ethanolamine-Bx (**8**), glutamic acid-Bx (**11**), *γ*-aminobutyric acid-Bx (**13**), proline-Bx (**14**), and phenylalanine-Bx (**22**), also identified in *B. vulgaris*, showed negative correlation against the same Gram-positive bacterial strains, confirming the effect of these compounds on their antimicrobial potential [33]. Glutamine-Bx (**5**) showed a positive effect against three fungal strains such as *C. albicans* ATCC 10231, *C. glabrata* ATCC 90,030, and *C. krusei* ATCC 14243. However, ten betaxanthins (**5**, **8**, **10**, **11**, **13**, **14**, **19**, **21′**, **21**, and **22**) found in *P. grandiflora* and *B. vulgaris* also showed a positive effect on activity against *C. albicans* ATCC 10231.

Recently, an interesting group of antimicrobial agents with strong activity against resistant bacteria and fungi are antimicrobial peptides (AMP) consisting of various amino acids. The designed sequence with strong antimicrobial activity against the gram-positive bacteria *S. aureus* ATCC 25923 and *E. faecalis* ATCC 29212 contains 24 amino acid residues, in which the lysine, histidine, and serine residues were replaced with arginine, and also the hydrophobic phenylalanine was replaced with tryptophan [54]. Such amino acids are present in the betaxanthins (**4** and **23**) in *B. vulgaris*, with a significant effect on the activity against these two strains of Gram-positive bacteria.

An amino acid inhibitor consisting of glutamic acid sulfonamides was also synthesized, which showed good inhibitory activity against *E. coli* ATCC 25922 [55]. In *B. vulgaris*, glutamic acid-Bx (**11**) consisting of the above amino acid also showed a strong effect on the activity against this strain of Gram-negative bacteria.

### 3.4. Cytotoxicity and Anticancer Selectivity

**Figure 6:**
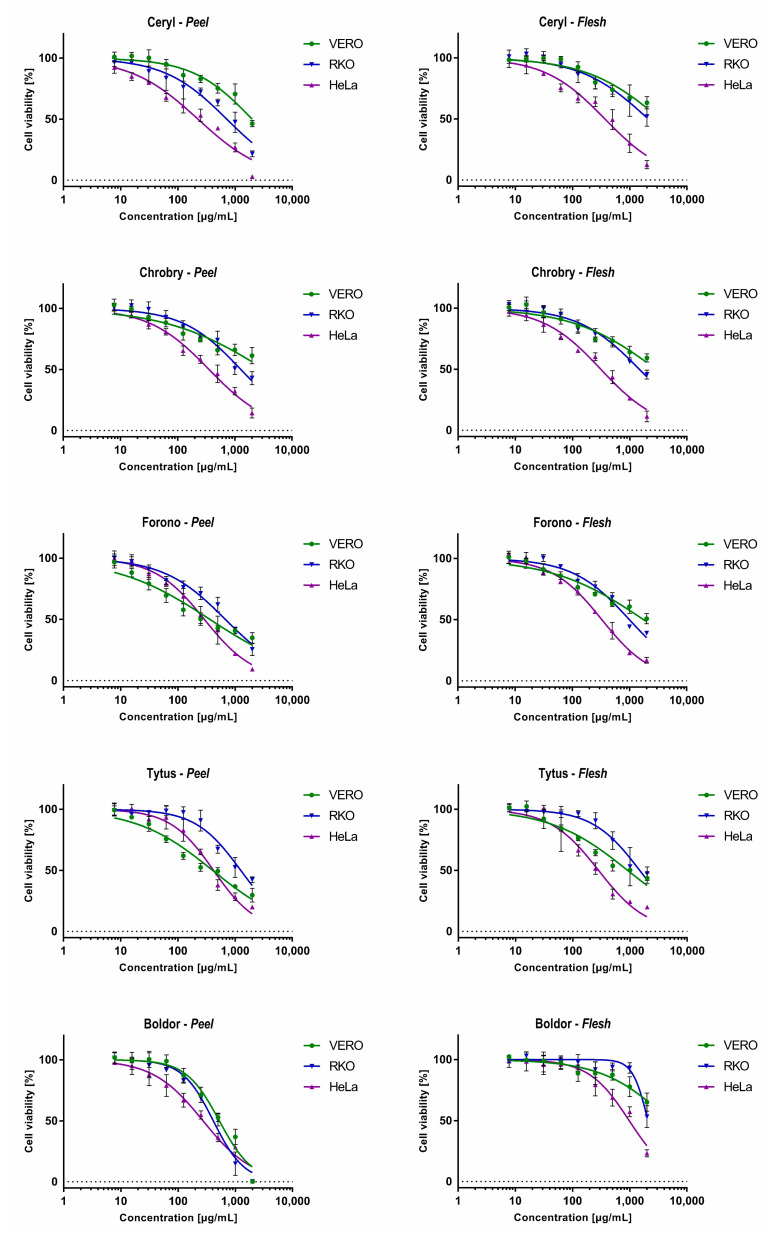
The dose-response impact of extracts from red and yellow *B. vulgaris* cultivars on non-cancerous and cancer cell lines.

The cytotoxicity was evaluated using an MTT-based assay towards non-cancerous VERO cells and cancer cell lines originating from colon cancer (RKO) and cervical adenocarcinoma (HeLa). The calculated CC_50_ (50% cytotoxic concentration) values are presented in Table 7, and the dose-response effects are in Figure 6.

Overall, the extracts obtained from the flesh of tested cultivars were less toxic to non-cancerous cells than the peel extracts obtained from the same plant material, except the Chrobry cultivar. Interestingly, the extracts of the peel and flesh of the Ceryl and Chrobry cultivars showed lower toxicity on VERO cells than those obtained from the Forono and Tytus cultivars. In the case of the Boldor cultivar, extracts from flesh showed the lowest observed toxicity among all tested samples with CC_50_ of 4948.6 μg/mL, and the extracts from peel were significantly more toxic (CC_50_ 527.7 μg/mL). Taking into account the classification of plant extracts cytotoxicity [56,57] extracts from tested cultivars can be regarded as non-cytotoxic (CC_50_ > 500 μg/mL) to VERO cells, except peel extracts from Forono and Tytus cultivars where weak cytotoxic activity (CC_50_ 201–500 μg/mL) was observed. The peel extract from the Boldor cultivar showed weak cytotoxicity towards both cancer cell lines without showing a cytotoxic effect on non-cancerous cells. HeLa cells were noticeably more sensitive to most of the extracts tested than VERO and RKO.

**Figure 7:**
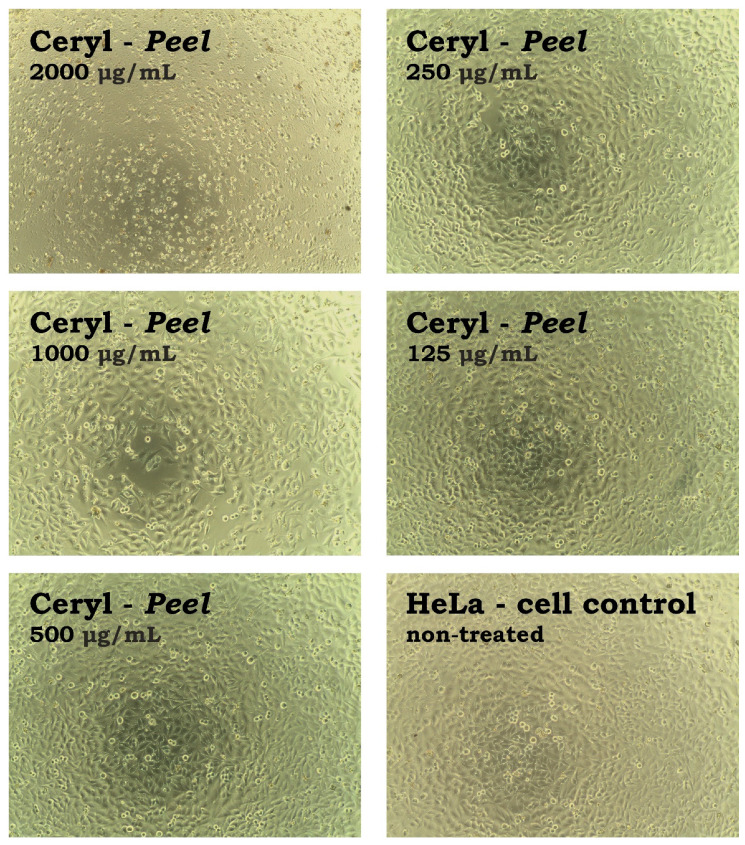
The influence of serial dilutions of extracts from the peel of *B. vulgaris* Ceryl cultivar on the cell line derived from cervical adenocarcinoma.

The anticancer potential was evaluated by comparing the CC_50_ values obtained on cancer cells with those observed on VERO cells. The calculated SI (selectivity index) is shown in Table 7. Extracts from the peel of Ceryl and Chrobry cultivars and flesh of Boldor cultivar showed potential selectivity towards colon cancer cells with SI between 2.37 and 2.78. Additionally, extracts from both the peel and flesh of the Ceryl and Chrobry cultivars showed highly selective cytotoxicity to cervical cancer cells with SI between 8.84 and 9.39. The influence of extract from the peel of the Ceryl cultivar on the morphology of the HeLa cell monolayer is presented in Figure 7.

At the concentration of 2000 μg/mL, the monolayer was destroyed, whereas, at half-lower concentration, there were some HeLa cells present, but the monolayer was noticeably less dense than the cell control. Further dilutions resulted in the increase of the confluence of the HeLa monolayer. Selectivity towards HeLa was also exerted by extracts from the flesh of Forono, Tytus, and Boldor cultivars (SI 3.22–5.35). Despite the potential anticancer selectivity, it must be underlined that this selectivity was observed at relatively high 50% cytotoxic concentrations. According to published literature [56,57], high cytotoxic activity and anticancer potential can be reported for plant extracts showing CC_50_ < 20 μg/mL.

Hence, the *B. vulgaris* extracts studied herein, despite showing potential selectivity towards cancer cell lines, failed to exert significant anticancer potential. Interestingly, Romero et al. [58] reported that the beetroot and leaf extracts decreased the viability of HeLa cells and potentiated the anticancer effects of cisplatin and rapamycin. Additionally, the beetroot extract 100 μg/mL induced early apoptosis in HeLa cells, decreased the cell size, and promoted cell death [58].

Clement et al. [59] reported that beetroot juice, or blended beetroot, was the second most popular functional food used by patients suffering from prostate, breast, and colorectal cancer, in combination with anticancer drugs [59]. Thanks to the multi-target action of *B. vulgaris* phytochemicals, including antioxidant activity, direct inhibition of proliferation, downregulation of the cancer pro-survival genes, ant-angiogenic activity, and inhibition of anti-apoptotic genes, they can be used in combination with conventional anticancer drugs to increase their efficacy and reduce toxicity, and overcome the multidrug resistance of cancer cells [60]. Moreover, *B. vulgaris* root extracts were shown to exert a chemopreventative role in cancer development [1,61,62].

### 3.5. Correlation between Phytochemical Composition and Anticancer Activity

Table 8 presents the correlation coefficients (R) calculated on the basis of the absolute peak area of each assigned peak from the chromatograms of the individual betaxanthins and the anticancer activity expressed by the CC_50_ parameter. The positive effect of the compounds on anticancer activity is represented by the negative value of the R coefficient, which refers to the decrease in the CC_50_ parameter with the increase in the component peak value (negative correlation).

The strongest positive effect on the activity against cancer cells derived from colon cancer (RKO) and cervical adenocarcinoma (HeLa) cell lines was shown by arginine-Bx (**4**), proline-Bx (**14**) and tryptophan-Bx (**23**). It should be noted that almost all identified betaxanthins in *B. vulgaris* exhibited a negative correlation against HeLa cell lines, with the exception of dopamine-Bx (**16**) and tyrosine-Bx (**17**). In contrast, negative correlation against RKO cell lines showed only ten betaxanthins such as asparagine-Bx (**2**), arginine-Bx (**4**), ornithine-Bx (**6**), ethanolamine-Bx (**8**), glutamic acid-Bx (**11**), *γ*-aminobutyric acid-Bx (**13**), proline -Bx (**14**), leucine-Bx (**21**), phenylalanine-Bx (**22**), and tryptophan-Bx (**23**).

Dopamine-Bx (**16**) and tyrosine-Bx (**17**) were the only ones to show a positive correlation against the two tested cell lines, RKO and HeLa, which suggests that they do not affect the anticancer activity against these cancer cells. Positive correlation against RKO cell lines was shown by as many as fourteen betaxanthins from *B. vulgaris*. In conclusion, identified betaxanthins definitely showed a stronger effect on anticancer activity against HeLa cell lines than against RKO cell lines.

According to the literature, among the betalains tested, tryptophan-Bx (**23**) showed the greatest effect on the size of the tumor by reducing its size by 56.4% in the animal model of *Caenorhabditis elegans* (cancer strain JK1466) and extended the animal’s lifespan by 9.3%, which indicates high effectiveness and low toxicity [63]. According to our results, tryptophan-Bx (**23**) showed strong activity against HeLa and RKO cell lines, so further in vitro and in vivo studies in this direction on the pure compound are also worth considering. It is worth noting that the above compound (**23**) may prove to be a potential chemotherapeutic with possible importance in chemoprevention and treatment strategies for colon cancer and cervical adenocarcinoma.

Arginine-Bx (**4**) is a product of betalamic acid with the amino acid (arginine). *L*-arginine is a substrate of nitric oxide synthase. Macrophages associated with early-stage cancer act to inhibit tumor growth by secreting nitric oxide. Nitric oxide produced from *L*-arginine by macrophages has been confirmed to work to enhance the effects of the anticancer drug (doxorubicin). This suggests that co-administration of *L*-arginine with doxorubicin would be an effective treatment to enhance chemotherapy [64]. Many studies confirm the anticancer activity of arginine and often use it as an additive to enhance the effects of potential anticancer drugs [64,65,66,67,68]. In *B. vulgaris*, arginine-Bx (**4**) showed a strong effect on the RKO and HeLa cell lines; therefore, it may also be a potential anticancer drug candidate like *L*-alanine.

According to the literature, proline-Bx (**14**), more popular under the name of indicaxanthin, can act as a neuromodulator, anti-inflammatory and anticancer agent in the prevention or treatment of neurological diseases and related cancer pathologies [69]. Combining phytochemicals with chemotherapeutic agents has become very popular recently as a new cancer treatment strategy to overcome drug toxicity and resistance to natural compounds. It has also been shown that proline-Bx (**14**) individually exhibits growth inhibitory effects on HeLa cervical cancer cells and in combination with cisplatin, collaborates in producing enhanced anticancer activity against these cells [2]. In conclusion, these studies confirm previous reports on the anticancer activity of this compound.

## 4. Conclusions

Obtained results enabled the characterization of 24 betaxanthins in all studied samples, of which two new betaxanthins (arginine-Bx and ornithine-Bx) were detected for the first time in *Beta vulgaris* L. cultivars. Glutamine-Bx, isoleucine-Bx, leucine-Bx, *γ*-aminobutyric acid-Bx, proline-Bx, and valine-Bx were dominant compounds in the cultivars studied. The content of betaxanthins was noticed in the peel and flesh of five cultivars of *B. vulgaris* for the first time and decreased in the following order, depending on the cultivar: Tytus > Ceryl > Chrobry > Forono > Boldor. The highest total content of betaxanthins (1231 mg/100 g Dry Extract) was observed for the Tytus (peel) cultivar. The Boldor (flesh) accumulated the lowest betaxanthin content (317 mg/100 g Dry Extract). Importantly, the peel of *B. vulgaris*, which are often considered waste, appeared to be a richer source of betaxanthins compared to its flesh, indicating wastes as a valuable and rich source of active compounds.

Tytus and Chrobry showed a moderate or good bactericidal effect, especially against the majority of Gram-positive bacteria. Ceryl peel extract was most active against *Staphylococcus aureus* and *Micrococcus luteus* at minimal inhibitory concentration (MIC) = 0.06 mg/mL and fungi with MIC = 0.25–0.5 mg/mL and was not cytotoxic (CC_50_ =1992 μg/mL, CC_50_ – 50% cytotoxic concentration). Forono peel showed the highest activity against *Micrococcus luteus* and *Bacillus subtilis* at MIC = 0.06 mg/mL. The good and moderate activity against most of the tested fungi was shown by the peel of Tytus and Ceryl (MIC = 0.125–0.5 mg/mL).

It should be noted that Gram-positive bacteria were more sensitive to the antibacterial effect of beetroot extracts, which is related to the structure of their cell wall consisting of a thick permeable layer of murein. However, it was noted that Gram-negative bacteria were resistant to the antimicrobial effect of beetroot extracts. This may be related not only to the content of active substances in the tested extracts but may also be influenced by the external structure of this group of bacteria. Namely, the cell wall of gram-negative bacteria consists of a thin layer of murein, over which there is an additional thick impermeable outer membrane made of phospholipids and hydrophilic lipopolysaccharides. This makes it difficult for extracts to overcome such a barrier of macromolecules and hydrophobic compounds.

All identified betaxanthins might have possessed bactericidal effect against one strain of Gram-positive bacteria *Staphylococcus epidermidis* ATCC 12228 and two strains of Gram-negative bacteria *Proteus mirabilis* ATCC 12453 and *Klebsiella pneumoniae* ATCC 13883, respectively. Asparagine-Bx and phenylalanine-Bx might be active against all bacteria and yeast tested. The remaining betaxanthins were sensitive to selected strains.

*B. vulgaris* extracts are safe and has weak or no cytotoxic activity. Our results indicate that studied extracts show stronger activity against cervical adenocarcinoma (HeLa) cells than the colon cancer cell line (RKO). Similarly, betaxanthins show a stronger effect on anticancer activity against HeLa cell lines than against RKO cell lines. Arginine-Bx, proline-Bx and tryptophan-Bx might be considered as betaxanthins with the highest anticancer potential due to the strongest activity against RKO and HeLa cell lines, what was indicated by correlation analysis.

Studies indicate that betaxanthins have significant potential application, so it is worth continuing research on selected compounds.

## Figures and Tables

**Table 1:** Chromatographic, spectrophotometric, and mass-spectrometric data of analyzed betaxanthins in fresh *Beta vulgaris* L. var. Ceryl, Chrobry, Forono, Tytus, Boldor.

No.	Betaxanthins	Trivial Name	t_R_ (min)	λ_max_ (nm)	*m/z* (M + H)^+^
1	Histidine-Bx	muscaaurin VII	4.4	470	349
2	Asparagine-Bx	vulgaxanthin III	5.1	468	326
3	Serine-Bx		5.7	469	299
4	Arginine-Bx		6.3	468	368
5	Glutamine-Bx	vulgaxanthin I	6.6	467	340
6	Ornithine-Bx		7.1	465	326
7	Glycine-Bx		7.2	470	269
8	Ethanolamine-Bx		7.5	454	255
9	Lysine-Bx		7.6	458	340
10	Threonine-Bx		8.7	469	313
11	Glutamic acid-Bx	vulgaxanthin II	9.2	469	341
12	Alanine-Bx		11.5	466	283
13	*γ*-Aminobutyric acid-Bx		11.6	454	297
14	Proline-Bx	indicaxanthin	14.6	477	309
15	Dopa-Bx	dopaxanthin	16.1	470	391
16	Dopamina-Bx	miraxanthin V	18.2	460	347
17	Tyrosine-Bx	portulacaxanthin II	18.4	471	375
18	Methionine-Bx		19.2	468	343
19	Valine-Bx		19.6	469	311
20	3-methoxytyramine-Bx		20.0	471	361
21	Isoleucine-Bx		22.0	469	325
21	Leucine-Bx	vulgaxanthin IV	22.2	469	325
22	Phenylalanine-Bx		22.3	469	359
23	Tryptophan-Bx	vulgaxanthin IV	22.3	473	398

**Table 2:** Content of individual betaxanthins and total betaxanthins in the peel of red and yellow *Beta vulgaris* L. cultivars (mg/100 g of Dry Extract) analyzed by LC-DAD-ESI-MS.

		Mass of Betaxanthins (mg) in 100 g of Dry Extract (DE) *Beta vulgaris* L.				
		Red Cultivar				Yellow Cultivar
		Ceryl	Chrobry	Forono	Tytus	Boldor
No.	Betaxanthins					
1	Histidine-Bx	0.15 ± 0.01 ^e^	1.06 ± 0.08 ^c^	0.88 ± 0.05 ^d^	1.87 ± 0.14 ^b^	1.93 ± 0.14 ^a^
2	Asparagine-Bx	42.52 ± 2.6 ^a^	26.91 ± 1.8 ^b^	24.40 ± 1.5 ^c^	42.52 ± 2.6 ^a^	11.12 ± 0.80 ^d^
3	Serine-Bx	23.81 ± 1.8 ^a^	16.28 ± 1.3 ^c^	9.72 ± 0.74 ^d^	19.64 ± 1.7 ^b^	5.94 ± 0.47 ^e^
4	Arginine-Bx	10.35 ± 0.68 ^d^	5.64 ± 0.37 ^e^	15.61 ± 1.0 ^c^	19.50 ± 1.5 ^b^	23.71 ± 1.5 ^a^
5	Glutamine-Bx	264.95 ± 0.88 ^b^	257.82 ± 15 ^c^	208.23 ± 16 ^d^	350.82 ± 21 ^a^	93.35 ± 6.1 ^e^
6	Ornithine-Bx	8.68 ± 0.57 ^e^	11.99 ± 0.79 ^d^	27.87 ± 1.7 ^c^	39.69 ± 2.7 ^a^	36.31 ± 2.4 ^b^
7	Glycine-Bx	3.28 ± 0.24 ^b^	1.91 ± 0.12 ^d^	2.51 ± 0.17 ^c^	7.72 ± 0.5 ^a^	1.64 ± 0.14 ^e^
8	Ethanolamine-Bx	10.07 ± 0.68 ^b^	5.26 ± 0.34 ^e^	6.55 ± 1.1 ^d^	10.83 ± 0.8 ^a^	8.53 ± 0.60 ^c^
9	Lysine-Bx	1.26 ± 0.11 ^c^	0.80 ± 0.061 ^e^	0.96 ± 0.06 ^d^	12.41 ± 0.87 ^a^	3.10 ± 0.21 ^b^
10	Threonine-Bx	12.17 ± 0.81 ^e^	20.58 ± 1.4 ^c^	15.88 ± 1.0 ^d^	35.43 ± 2.4 ^a^	20.70 ± 1.5 ^b^
11	Glutamic acid-Bx	56.49 ± 4.8 ^a^	25.46 ± 1.7 ^c^	18.25 ± 1.3 ^d^	56.54 ± 3.5 ^a^	28.09 ± 1.8 ^b^
12	Alanine-Bx	14.23 ± 0.91 ^b^	10.87 ± 0.82 ^c^	9.40 ± 0.68 ^e^	21.59 ± 1.8 ^a^	10.57 ± 0.8 ^d^
13	*γ*-Aminobutyric acid-Bx	66.03 ± 5.1 ^b^	35.14 ± 2.1 ^e^	36.88 ± 2.8 ^d^	70.06 ± 4.8 ^a^	50.32 ± 3.1 ^c^
14	Proline-Bx	65.79 ± 5.4 ^c^	35.70 ± 2.3 ^e^	52.97 ± 4.1 ^d^	82.53 ± 5.2 ^b^	140.95 ± 10^a^
15	Dopa-Bx	0.21 ± 0.01 ^a^	0.11 ± 0.008 ^b^	0.05 ± 0.004 ^c^	0.30 ± 0.02 ^a^	0.03 ± 0.002 ^c^
16	Dopamine-Bx	0.13 ± 0.009 ^d^	0.21 ± 0.014 ^c^	0.04 ± 0.003 ^e^	2.36 ± 0.17 ^a^	0.39 ± 0.03 ^b^
17	Tyrosine-Bx	0.10 ± 0.006 ^e^	1.01 ± 0.074 ^c^	1.08 ± 0.07 ^b^	1.65 ± 0.12 ^a^	0.25 ± 0.02 ^d^
18	Methionine-Bx	7.81 ± 0.63 ^a^	3.85 ± 0.25 ^c^	3.05 ± 0.23 ^d^	4.57 ± 0.32 ^b^	0.99 ± 0.06 ^e^
19	Valine-Bx	55.61 ± 4.6 ^b^	45.22 ± 2.8 ^d^	45.52 ± 2.8 ^c^	74.29 ± 4.5 ^a^	40.69 ± 2.8 ^e^
20	3-methoxytyramine-Bx	27.82 ± 1.8 ^b^	14.52 ± 1.3 ^d^	15.47 ± 1.3 ^c^	34.14 ± 2.6 ^a^	3.40 ± 0.26 ^e^
21′	Isoleucine-Bx	115.09 ± 7.1 ^b^	102.17 ± 6.4 ^c^	74.83 ± 5.4 ^d^	159.96 ± 10 ^a^	34.14 ± 2.3 ^e^
21	Leucine-Bx	100.32 ± 6.8 ^b^	74.91 ± 5.2 ^c^	74.38 ± 4.8 ^d^	150.47 ± 9.7 ^a^	32.62 ± 2.1 ^e^
22	Phenylalanine-Bx	20.58 ± 1.5 ^a^	13.86 ± 0.8 ^c^	15.00 ± 1.0 ^b^	20.75 ± 1.5 ^a^	4.16 ± 0.35 ^d^
23	Tryptophan-Bx	12.05 ± 0.83 ^c^	7.28 ± 0.57 ^e^	9.75 ± 0.78 ^d^	12.23 ± 0.78 ^b^	21.37 ± 1.4 ^a^
**Total concentration**		**919** ± **52 ^b^**	**718** ± **36 ^c^**	**669** ± **28 ^d^**	**1231** ± **64 ^a^**	**574 ± 26 ^e^**

The superscript letters within each row (^a–e^) mean significant differences between results (*p* < 0.05).

**Table 3:** Content of individual betaxanthins and total betaxanthins in the flesh of red and yellow *Beta vulgaris* L. cultivars (mg/100 g of Dry Extract) analyzed by LC-DAD-ESI-MS.

		Mass of Betaxanthins (mg) in 100 g of Dry Extract (DE) *Beta vulgaris* L.				
		Red Cultivar				Yellow Cultivar
		Ceryl	Chrobry	Forono	Tytus	Boldor
No.	Betaxanthins					
1	Histidine-Bx	1.56 ± 0.09 ^c^	2.84 ± 0.21 ^a^	1.85 ± 0.16 ^b^	0.07 ± 0.005 ^e^	0.38 ± 0.02 ^d^
2	Asparagine-Bx	24.64 ± 1.8 ^a^	2.84 ± 0.18 ^e^	21.33 ± 1.4 ^c^	21.37 ± 1.5 ^b^	13.84 ± 0.90 ^d^
3	Serine-Bx	16.92 ± 1.1 ^c^	22.05 ± 1.7 ^a^	12.90 ± 0.84 ^d^	18.19 ± 1.2 ^b^	7.53 ± 0.55 ^e^
4	Arginine-Bx	2.11 ± 0.16 ^c^	2.80 ± 0.22 ^b^	3.71 ± 0.25 ^a^	2.31 ± 0.20 ^c^	0.51 ± 0.03 ^d^
5	Glutamine-Bx	179.79 ± 11 ^c^	219.64 ± 15 ^a^	172.67 ± 12 ^d^	190.90 ± 12 ^b^	80.10 ± 6.1 ^e^
6	Ornithine-Bx	3.84 ± 0.34 ^c^	3.24 ± 0.24 ^d^	6.81 ± 0.44 ^b^	3.91 ± 0.30 ^c^	11.37 ± 0.80 ^a^
7	Glycine-Bx	7.47 ± 0.56 ^c^	7.23 ± 0.44 ^d^	8.37 ± 0.68 ^b^	13.59 ± 0.95 ^a^	1.25 ± 0.08 ^e^
8	Ethanolamine-Bx	4.46 ± 0.27 ^b^	4.80 ± 0.29 ^a^	3.28 ± 0.26 ^c^	2.81 ± 0.21 ^d^	1.77 ± 0.12 ^e^
9	Lysine-Bx	0.61 ± 0.05 ^c^	0.35 ± 0.02 ^e^	0.49 ± 0.04 ^d^	4.91 ± 0.32 ^a^	4.25 ± 0.32 ^b^
10	Threonine-Bx	15.55 ± 1.1 ^d^	17.62 ± 1.2 ^a^	16.12 ± 1.4 ^c^	16.29 ± 1.2 ^b^	8.41 ± 0.65 ^e^
11	Glutamic acid-Bx	21.53 ± 1.4 ^a^	14.49 ± 0.98 ^d^	14.95 ± 1.2 ^c^	15.32 ± 0.97 ^b^	8.27 ± 0.58 ^e^
12	Alanine-Bx	15.90 ± 0.98 ^d^	24.96 ± 1.6 ^a^	16.65 ± 1.3 ^c^	23.46 ± 1.6 ^b^	11.34 ± 0.76 ^e^
13	*γ*-Aminobutyric acid-Bx	32.88 ± 2.3 ^a^	29.47 ± 2.0 ^b^	22.31 ± 1.6 ^d^	23.93 ± 1.7 ^c^	22.09 ± 0.22 ^e^
14	Proline -Bx	40.13 ± 2.6 ^c^	29.63 ± 2.0 ^d^	41.73 ± 2.6 ^b^	26.82 ± 1.9 ^e^	62.47 ± 4.2 ^a^
15	Dopa-Bx	0.08 ± 0.006 ^d^	0.21 ± 0.02 ^c^	0.34 ± 0.03 ^b^	0.45 ± 0.03 ^a^	0.20 ± 0.02 ^c^
16	Dopamine-Bx	0.12 ± 0.008 ^c^	0.24 ± 0.02 ^b^	0.10 ± 0.006 ^c^	0.12 ± 0.008 ^c^	0.65 ± 0.04 ^a^
17	Tyrosine-Bx	1.87 ± 0.15 ^d^	3.04 ± 0.24 ^c^	6.02 ± 0.43 ^b^	1.71 ± 0.14 ^d^	7.71 ± 0.52 ^a^
18	Methionine-Bx	8.66 ± 0.59 ^b^	11.50 ± 0.7 ^a^	5.08 ± 0.37 ^d^	6.17 ± 0.50 ^c^	0.14 ± 0.009 ^e^
19	Valine-Bx	42.70 ± 2.7 ^c^	48.81 ± 3.5 ^b^	50.66 ± 3.7 ^a^	39.35 ± 2.8 ^d^	30.69 ± 2.2 ^e^
20	3-methoxytyramine-Bx	16.00 ± 0.98 ^a^	9.61 ± 0.74 ^b^	6.70 ± 0.5 ^d^	9.42 ± 0.57 ^c^	6.66 ± 0.44 ^d^
21′	Isoleucine-Bx	83.54 ± 5.8 ^a^	80.24 ± 5.4 ^b^	68.30 ± 4.1 ^c^	55.96 ± 3.6 ^d^	17.49 ± 1.3 ^e^
21	Leucine-Bx	55.17 ± 3.7 ^b^	59.86 ± 3.7 ^a^	55.08 ± 3.5 ^c^	41.21 ± 3.2 ^d^	13.17 ± 0.87 ^e^
22	Phenylalanine-Bx	8.95 ± 0.77 ^b^	6.80 ± 0.54 ^c^	9.30 ± 0.61 ^a^	6.21 ± 0.43 ^d^	1.23 ± 0.09 ^e^
23	Tryptophan-Bx	6.43 ± 0.55 ^b^	7.33 ± 0.61 ^a^	3.95 ± 0.30 ^e^	4.46 ± 0.31 ^d^	5.89 ± 0.47 ^c^
**Total concentration**		**590** ± **32 ^b^**	**609** ± **40 ^a^**	**548** ± **28 ^c^**	**528** ± **34 ^d^**	**317** ± **22 ^e^**

The superscript letters within each row (^a–e^) mean significant differences between results (*p* < 0.05).

**Table 4:** Antimicrobial activity of extracts obtained from red and yellow *B. vulgaris* cultivars assessed as MIC (minimum inhibitory concentration) and MBC (minimum bactericidal concentration) against strains of Gram-positive bacteria and correlation coefficients between identified betaxanthins (absolute peak areas) and microbial activity (MIC values). Statistical significance is marked by font: boldface means 95% significance, normal font lack of significance at 95%.

		Gram-Positive Bacteria								
**Microorganism/** **Extract**		*Staphylococcus aureus* ATCC 29213	*Staphylococcus aureus* ATCC 25923	*Staphylococcus aureus* ATCC 43300	*Staphylococcus aureus* ATCC BAA-1707	*Staphylococcus epidermidis* ATCC 12228	*Entarococcus faecalis* ATCC 29212	*Micrococcus luteus* ATCC 10240	*Bacillus subtilis* ATCC 6633	*Bacillus cereus* ATCC 10876
		**MIC;MBC**								
Ceryl	Peel	1;4	0.06;0.5	4;4	4;4	4;4	1;8	0.06;0.06	0.125;8	16;16
	Flesh	16;16	16;16	16;16	16;16	4;4	4;8	4;8	16;16	16;16
Chrobry	Peel	0.125;8	0.125;4	0.25;4	0.25;8	0.25;1	0.5;8	0.125;0.25	0.125;8	1;8
	Flesh	16;16	16;16	16;16	16;16	4;4	16;16	2;8	16;16	16;16
Forono	Peel	16;16	4;4	4;8	4;4	2;4	16;16	0.06;0.06	0.06;8	4;8
	Flesh	16;16	4;4	16;16	16;16	2;4	16;16	1;2	0.125;8	8;8
Tytus	Peel	0.25;8	0.25;8	0.25;4	0.5;8	0.25;8	0.5;8	0.125;0.25	16;16	16;16
	Flesh	16;16	16;16	16;16	16;16	4;4	4;8	0.5;0.5	16;16	16;16
Boldor	Peel	16;16	16;16	16;16	16;16	16;16	4;8	4;8	16;16	4;8
	Flesh	16;16	16;16	16;16	16;16	16;16	4;8	2;4	16;16	16;16
		**Correlation**								
**No.**	**Betaxanthins**									
1	Histidine-Bx	0.274	0.274	0.307	0.308	**−0.306**	0.597	0.328	**0.127**	0.223
2	Asparagine-Bx	**−0.453**	**−0.543**	**−0.413**	**−0.414**	**−0.629**	**−0.287**	**−0.398**	**−0.451**	**−0.049**
3	Serine-Bx	**−0.086**	**0.009**	**0.078**	**0.077**	**−0.592**	***0.109***	**−0.131**	**−0.029**	0.362
4	Arginine-Bx	**−0.368**	**−0.587**	**−0.646**	**−0.645**	**−0.259**	**0.026**	**−0.349**	**−0.363**	**−0.459**
5	Glutamine-Bx	**−0.307**	**−0.346**	**−0.276**	**−0.276**	**−0.870**	**0.134**	**−0.407**	**−0.275**	**0.159**
6	Ornithine-Bx	**−0.307**	**−0.547**	**−0.651**	**−0.648**	**−0.281**	**0.046**	**−0.344**	**−0.236**	**−0.376**
7	Glycine-Bx	0.343	0.326	0.453	0.455	**−0.383**	0.206	**−0.001**	0.253	0.527
8	Ethanolamine-Bx	**−0.513**	**−0.524**	**−0.510**	**−0.511**	**−0.574**	**−0.075**	**−0.300**	**−0.352**	**−0.091**
9	Lysine-Bx	**−0.304**	**−0.108**	**−0.240**	**−0.233**	**−0.236**	**−0.416**	**−0.347**	0.411	0.450
10	Threonine-Bx	**−0.124**	**−0.166**	**−0.108**	**−0.105**	**−0.818**	0.197	**−0.184**	**0.015**	0.253
11	Glutamic acid-Bx	**−0.655**	**−0.554**	**−0.504**	**−0.504**	**−0.508**	**−0.368**	**−0.340**	**−0.308**	**0.002**
12	Alanine-Bx	0.234	0.268	0.363	0.364	**−0.479**	0.315	**−0.023**	0.215	0.526
13	*γ*-Aminobutyric acid-Bx	**−0.507**	**−0.459**	**−0.437**	**−0.438**	**−0.586**	**−0.177**	**−0.274**	**−0.283**	**0.026**
14	Proline-Bx	**−0.178**	**−0.314**	**−0.172**	**−0.171**	**−0.223**	**−0.019**	**0.113**	**−0.240**	**−0.235**
15	Dopa-Bx	**0.135**	**0.065**	0.296	0.297	**−0.380**	0.182	**−0.329**	**0.028**	0.373
16	Dopamine-Bx	**−0.515**	**−0.318**	**−0.433**	**−0.424**	**−0.279**	**−0.327**	**−0.222**	0.356	0.384
17	Tyrosine-Bx	0.451	**0.123**	0.518	0.519	**−0.219**	0.643	0.067	**−0.091**	0.247
18	Methionine-Bx	**0.171**	0.248	0.306	0.305	**−0.426**	0.288	**0.153**	**0.130**	0.443
19	Valine-Bx	**−0.097**	**−0.256**	**−0.052**	**−0.052**	**−0.789**	0.321	**−0.238**	**−0.259**	0.193
20	3-methoxytyramine-Bx	**−0.552**	**−0.483**	**−0.515**	**−0.514**	**−0.652**	**−0.265**	**−0.338**	**−0.233**	**0.152**
21′	Isoleucine-Bx	**−0.426**	**−0.435**	**−0.359**	**−0.359**	**−0.840**	**0.015**	**−0.306**	**−0.280**	**0.141**
21	Leucine-Bx	**−0.527**	**−0.608**	**−0.528**	**−0.527**	**−0.857**	**0.032**	**−0.474**	**−0.373**	**0.060**
22	Phenylalanine-Bx	**−0.555**	**−0.715**	**−0.607**	**−0.609**	**−0.774**	**−0.029**	**−0.541**	**−0.614**	**−0.181**
23	Tryptophan-Bx	**−0.273**	**−0.241**	**−0.312**	**−0.313**	**−0.307**	**−0.017**	**0.015**	**−0.192**	**−0.212**

MIC and MBC were expressed as mg/mL. The representative data (mode) are presented.

**Table 5:** Antimicrobial activity of extracts obtained from red and yellow *B. vulgaris* cultivars assessed as MIC (minimum inhibitory concentration) and MBC (minimum bactericidal concentration) against strains of Gram-negative bacteria and correlation coefficients between identified betaxanthins (absolute peak areas) and microbial activity (MIC values). Statistical significance is marked by font: boldface means 95% significance, and normal font lack of significance at 95%.

		Gram-Negative Bacteria					
**Microorganism/** **Extract**		*Salmonella* Typhimurium ATCC 14028	*Proteus mirabilis* ATCC 12453	*Bordetella bronchiseptica* ATCC 4617	*Escherichia coli* ATCC 25922	*Klebsiella pneumoniae* ATCC 13883	*Pseudomonas aeruginosa* ATCC 27853
		**MIC;MBC**					
Ceryl	Peel	16;16	16;16	2;8	4;8	16;16	4;8
	Flesh	16;16	16;16	16;16	16;16	16;16	4;8
Chrobry	Peel	4;8	8;8	2;4	4;4	16;16	2;8
	Flesh	16;16	16;16	4;8	16;16	16;16	4;8
Forono	Peel	16;16	16;16	2;4	4;4	16;16	4;4
	Flesh	16;16	16;16	8;8	16;16	16;16	4;8
Tytus	Peel	16;16	16;16	4;4	4;8	16;16	4;8
	Flesh	16;16	16;16	4;8	4;8	16;16	4;8
Boldor	Peel	16;16	16;16	8;8	16;16	16;16	4;8
	Flesh	16;16	32;32	16;16	16;16	32;32	4;8
		**Correlation**					
**No.**	**Betaxanthins**						
1	Histidine-Bx	**0.096**	**−0.34**	**0.092**	0.540	**−0.343**	**0.096**
2	Asparagine-Bx	**−0.025**	**−0.46**	**−0.228**	**−0.538**	**−0.462**	**−0.025**
3	Serine-Bx	**0.068**	**−0.51**	**−0.246**	**−0.129**	**−0.515**	**0.068**
4	Arginine-Bx	**0.137**	**−0.48**	**−0.585**	**−0.531**	**−0.477**	**0.137**
5	Glutamine-Bx	**−0.084**	**−0.67**	**−0.474**	**−0.403**	**−0.673**	**−0.084**
6	Ornithine-Bx	**0.041**	**−0.34**	**−0.452**	**−0.495**	**−0.344**	**0.041**
7	Glycine-Bx	0.279	**−0.36**	**0.012**	**0.023**	**−0.359**	0.279
8	Ethanolamine-Bx	**0.099**	**−0.63**	**−0.501**	**−0.442**	**−0.632**	**0.099**
9	Lysine-Bx	0.194	**−0.14**	**−0.233**	**−0.508**	**−0.138**	0.194
10	Threonine-Bx	**−0.055**	**−0.73**	**−0.320**	**−0.205**	**−0.731**	**−0.055**
11	Glutamic acid-Bx	**0.042**	**−0.48**	**−0.380**	**−0.488**	**−0.485**	**0.042**
12	Alanine-Bx	0.232	**−0.45**	**−0.140**	**0.053**	**−0.450**	0.232
13	*γ*-Aminobutyric acid -Bx	**0.102**	**−0.61**	**−0.414**	**−0.440**	**−0.609**	**0.102**
14	Proline-Bx	0.345	**−0.66**	**−0.202**	**−0.091**	**−0.660**	0.345
15	Dopa-Bx	0.205	**−0.28**	**−0.240**	**−0.146**	**−0.281**	0.205
16	Dopamine-Bx	**0.094**	**−0.10**	**−0.145**	**−0.263**	**−0.103**	**0.094**
17	Tyrosine-Bx	0.202	**−0.02**	0.252	0.516	**−0.021**	0.202
18	Methionine-Bx	0.188	**−0.42**	**−0.019**	0.182	**−0.421**	0.188
19	Valine-Bx	**0.117**	**−0.68**	**−0.310**	**−0.147**	**−0.678**	**0.117**
20	3-methoxytyramine-Bx	**0.051**	**−0.47**	**−0.299**	**−0.511**	**−0.470**	**0.051**
21′	Isoleucine-Bx	**−0.082**	**−0.65**	**−0.348**	**−0.335**	**−0.645**	**−0.082**
21	Leucine-Bx	**−0.011**	**−0.65**	**−0.499**	**−0.481**	**−0.647**	**−0.011**
22	Phenylalanine-Bx	**−0.082**	**−0.58**	**−0.509**	**−0.576**	**−0.576**	**−0.082**
23	Tryptophan-Bx	**0.155**	**−0.69**	**−0.449**	**−0.264**	**−0.694**	**0.155**

MIC and MBC were expressed as mg/mL. The representative data (mode) are presented.

**Table 6:** Antimicrobial activity of extracts obtained from red and yellow *B. vulgaris* cultivars assessed as MIC (minimum inhibitory concentration) and MFC (minimum fungicidal concentration) against *Candida* species and correlation coefficients between identified betaxanthins (absolute peak areas) and microbial activity (MIC values). Statistical significance is marked by font: boldface means 95% significance, and normal font lack of significance at 95%.

		Fungal (Yeasts) Strains							
**Microorganism/** **Extract**		*Candida parapsilosis* ATCC 22019	*Candida albicans* ATCC 10231	*Candida albicans* ATCC 2091	*Candida glabrata* ATCC 90030	*Candida krusei* ATCC 14243	*Candida auris* CDC B11903	*Candida lusitaniae* ATCC 3449	*Candida tropicalis* ATCC 1369
		**MIC;MFC**							
Ceryl	Peel	0.25;1	0.25;1	0.25;1	0.5;2	0.5;4	0.5;1	1;4	1;4
	Flesh	4;8	4;8	4;8	4;8	4;8	4;8	4;4	4;4
Chrobry	Peel	2;8	1;8	1;8	4;8	2;8	1;8	2;8	2;8
	Flesh	4;4	4;8	4;8	4;8	4;8	4;8	4;4	4;4
Forono	Peel	0.5;2	1;4	1;4	4;4	1;4	2;4	2;4	4;4
	Flesh	4;8	4;8	4;8	8;8	4;4	8;8	8;4	8;4
Tytus	Peel	0.25;2	0.25;2	0.25;2	1;4	0.25;0.5	0.5;2	0.125;4	0.25;4
	Flesh	8;8	4;4	4;4	8;8	4;4	8;8	4;4	4;4
Boldor	Peel	4;8	4;8	4;8	4;8	4;8	4;8	4;8	2;4
	Flesh	4;4	4;4	4;4	4;8	4;4	4;4	4;4	4;4
		**Correlation**							
**No.**	**Betaxanthins**								
1	Histidine-Bx	**0.066**	**0.066**	0.344	**0.149**	0.335	0.194	0.405	0.410
2	Asparagine-Bx	**−0.279**	**−0.494**	**−0.494**	**−0.165**	**−0.497**	**−0.168**	**−0.260**	**−0.126**
3	Serine-Bx	0.181	**0.019**	**0.019**	**0.059**	**0.024**	**0.140**	**0.020**	**0.091**
4	Arginine-Bx	**−0.719**	**−0.717**	**−0.717**	**−0.513**	**−0.780**	**−0.563**	**−0.572**	**−0.470**
5	Glutamine-Bx	**−0.109**	**−0.308**	**−0.308**	**−0.033**	**−0.299**	**−0.062**	**−0.164**	**0.010**
6	Ornithine-Bx	**−0.653**	**−0.648**	**−0.648**	**−0.378**	**−0.706**	**−0.497**	**−0.520**	**−0.381**
7	Glycine-Bx	0.678	0.441	0.441	0.621	0.411	0.703	0.418	0.451
8	Ethanolamine-Bx	**−0.590**	**−0.631**	**−0.631**	**−0.588**	**−0.661**	**−0.526**	**−0.517**	**−0.418**
9	Lysine-Bx	**0.105**	**−0.240**	**−0.240**	**−0.056**	**−0.299**	**0.012**	**−0.386**	**−0.391**
10	Threonine-Bx	**0.096**	**−0.065**	**−0.065**	0.198	**−0.069**	**0.156**	**0.029**	**0.128**
11	Glutamic acid-Bx	**−0.495**	**−0.627**	**−0.627**	**−0.573**	**−0.641**	**−0.474**	**−0.511**	**−0.475**
12	Alanine-Bx	0.500	0.338	0.338	0.402	0.316	0.497	0.297	0.343
13	*γ*-Aminobutyric acid-Bx	**−0.460**	**−0.558**	**−0.558**	**−0.517**	**−0.583**	**−0.432**	**−0.473**	**−0.391**
14	Proline-Bx	**−0.395**	**−0.299**	**−0.299**	**−0.338**	**−0.362**	**−0.208**	**−0.143**	**−0.201**
15	Dopa-Bx	0.575	0.267	0.267	0.604	0.248	0.675	0.393	0.413
16	Dopamine-Bx	**−0.358**	**−0.412**	**−0.412**	**−0.459**	**−0.465**	**−0.381**	**−0.489**	**−0.506**
17	Tyrosine-Bx	0.387	0.538	0.538	0.666	0.522	0.709	0.836	0.883
18	Methionine-Bx	0.258	0.255	0.255	**0.110**	0.245	0.224	0.187	0.236
19	Valine-Bx	**−0.025**	**−0.104**	**−0.104**	**0.116**	**−0.123**	**0.149**	**0.116**	0.255
20	3-methoxytyramine-Bx	**−0.483**	**−0.611**	**−0.611**	**−0.535**	**−0.636**	**−0.481**	**−0.553**	**−0.402**
21′	Isoleucine-Bx	**−0.298**	**−0.400**	**−0.400**	**−0.236**	**−0.395**	**−0.234**	**−0.243**	**−0.097**
21	Leucine-Bx	**−0.488**	**−0.593**	**−0.593**	**−0.362**	**−0.611**	**−0.362**	**−0.382**	**−0.200**
22	Phenylalanine-Bx	**−0.577**	**−0.693**	**−0.693**	**−0.381**	**−0.697**	**−0.423**	**−0.406**	**−0.201**
23	Tryptophan-Bx	**−0.447**	**−0.426**	**−0.426**	**−0.534**	**−0.458**	**−0.463**	**−0.447**	**−0.444**

MIC and MFC were expressed as mg/mL. The representative data (mode) are presented.

**Table 7:** Cytotoxicity and anticancer selectivity of *B. vulgaris* extracts on different cell lines after 24 h incubation.

Extract		VERO	RKO		HeLa	
		CC_50_	CC_50_	SI	CC_50_	SI
Ceryl	Peel	1992.00 ± 249.10	717.20 ± 79.32	2.78	225.30 ± 21.28	8.84
	Flesh	3432.04 ± 631.00	2100.23 ± 352.70	1.63	367.92 ± 33.30	9.33
Chrobry	Peel	3330.00 ± 865.15	1275.75 ± 124.20	2.61	354.80 ± 22.64	9.39
	Flesh	2896.15 ± 533.50	1470.35 ± 139.70	1.97	312.21 ± 19.80	9.28
Forono	Peel	361.10 ± 42.17	674.20 ± 51.81	0.54	290.23 ± 20.58	1.24
	Flesh	1754.31 ± 253.72	993.10 ± 76.91	1.77	328.10 ± 18.56	5.35
Tytus	Peel	405.40 ± 36.47	1259.00 ± 101.90	0.32	420.90 ± 29.37	0.96
	Flesh	908.45 ± 110.90	1479.66 ± 155.60	0.61	282.38 ± 25.24	3.22
Boldor	Peel	527.70 ± 35.85	413.80 ± 26.25	1.28	274.74 ± 22.14	1.92
	Flesh	4948.60 ± 1440.00	2090.00 ± 98.99	2.37	980.70 ± 74.37	5.05

CC_50_—50% cytotoxic concentration (mean ± SD) μg/mL; VERO—non-cancerous cells; RKO—colon cancer cell line; HeLa—cervical adenocarcinoma; SI—selectivity index (CC_50_VERO/CC_50_CancerCells).

**Table 8:** Correlation coefficients between identified betaxanthins (absolute peak areas) and microbial activity (CC_50_). Statistical significance is marked by font: boldface means 95% significance, and normal font lack of significance at 95%.

		RKO	HeLa
No.	Betaxanthins		
1	Histidine-Bx	0.177	**−0.242**
2	Asparagine-Bx	**−0.080**	**−0.453**
3	Serine-Bx	0.211	**−0.520**
4	Arginine-Bx	**−0.771**	**−0.490**
5	Glutamine-Bx	**0.044**	**−0.628**
6	Ornithine-Bx	**−0.575**	**−0.274**
7	Glycine-Bx	0.320	**−0.334**
8	Ethanolamine-Bx	**−0.357**	**−0.650**
9	Lysine-Bx	**0.068**	**−0.044**
10	Threonine-Bx	**0.113**	**−0.593**
11	Glutamic acid-Bx	**−0.221**	**−0.499**
12	Alanine-Bx	0.282	**−0.430**
13	*γ*-Aminobutyric acid-Bx	**−0.220**	**−0.622**
14	Proline-Bx	**−0.574**	**−0.689**
15	Dopa-Bx	**0.089**	**−0.300**
16	Dopamine-Bx	**0.075**	**0.074**
17	Tyrosine-Bx	0.242	**0.030**
18	Methionine-Bx	0.318	**−0.416**
19	Valine-Bx	**0.010**	**−0.636**
21′	Isoleucine-Bx	**0.059**	**−0.571**
21	Leucine-Bx	**−0.130**	**−0.585**
22	Phenylalanine-Bx	**−0.265**	**−0.573**
23	Tryptophan-Bx	**−0.498**	**−0.753**

CC_50_—50% cytotoxic concentration μg/mL; RKO—colon cancer cell line; HeLa—cervical adenocarcinoma.

## Data Availability

Data is contained within the article.
